# Serotypes, Antimicrobial Susceptibility, and Potential Mechanisms of Resistance Gene Transfer in *Erysipelothrix rhusiopathiae* Strains from Waterfowl in Poland

**DOI:** 10.3390/ijms252212192

**Published:** 2024-11-13

**Authors:** Marta Dec, Tomasz Nowak, John Webster, Karolina Wódz

**Affiliations:** 1 Department of Veterinary Prevention and Avian Diseases, Faculty of Veterinary Medicine, University of Life Sciences in Lublin, Akademicka 12, 20-033 Lublin, Poland; 2 Laboratory of Molecular Biology, Vet-Lab Brudzew, Turkowska 58c, 62-720 Brudzew, Poland; tomasz@labbrudzew.pl (T.N.); karolina.wodz@labbrudzew.pl (K.W.); 3 Elizabeth Macarthur Agricultural Institute, NSW Department of Primary Industries and Regional Development, PMB 408, Narellan, NSW 2570, Australia; john.webster@dpi.nsw.gov.au

**Keywords:** erysipelas, waterfowl, geese, antimicrobial susceptibility, serotype

## Abstract

Erysipelas is a significant problem in the waterfowl farming in Poland, and information on the characteristics of the *Erysipelothrix rhusiopathiae* strains causing this disease is limited. In this study, we determined the serotypes, antimicrobial susceptibility, and potential mechanisms of resistance gene transfer in *E. rhusiopathiae* isolates (n = 60) from domestic geese and ducks. We also developed a multiplex PCR for the detection of resistance genes. The antimicrobial susceptibility of the isolates was assessed using the broth microdilution method. Resistance genes, integrative conjugative element (ICE)-specific genes, phage-specific genes, and serotype determinants were detected by PCR. Multilocus sequence typing (MLST) was performed for selected resistant strains. The comparative analyses included 260 *E. rhusiopathiae* strains whose whole genome sequences (WGSs) are publicly available. *E. rhusiopathiae* isolates represented 7 serotypes, among which serotypes 5 (38.3%) and 1b (28.3%) were the most common. All strains were susceptible to β-lactams, and the vast majority of them were resistant to tetracycline (85%) and enrofloxacin (80%). The percentages of isolates resistant to other antimicrobials used ranged from 3.3% to 16.7%. Ten isolates (16.7%) were found to be multidrug resistant (MDR). The genotypic resistance profiles of the *E. rhusiopathiae* strains corresponded to their phenotypic resistance, and the amplification patterns obtained using the 10-plex PCR developed in this study were fully consistent with the results of single PCRs. The most prevalent resistance gene was *tetM*. In enrofloxacin-resistant strains, nonsynonymous mutations in the *gyrA* and *parC* genes were identified. The presence of ICE-specific genes was confirmed in resistant strains, and in MDR isolates of serotype 8 that represented sequence type (ST) 113, prophage DNA (Javan630-like) linked to the *lsaE* gene was additionally detected. The results indicate that β-lactam antibiotics should be the first choice for the treatment of waterfowl erysipelas in Poland. ICEs, including a transposon from the Tn916/Tn1545 family, and bacteriophages are most likely responsible for the transfer of resistance genes in *E. rhusiopathiae*.

## 1. Introduction

*Erysipelothrix rhusiopathiae* (*E. rhusiopathiae*) is a facultative anaerobic, thin, Gram-positive, non-motile, and non-spore-forming rod-shaped bacterium that causes erysipelas in various animal species, as well as in humans [1]. It belongs to the genus *Erysipelothrix*, which currently includes 13 species: *E. rhusiopathiae*, *E. tonsillarum*, *E. inopinata*, *Erysipelothrix* sp. strain 1, *E. piscisicarius* (formerly *Erysipelothrix* sp. strain 2), *Erysipelothrix* sp. strain 3, *E. larvae*, *E. anatis*, *E. urinaevulpis*, *E. aquatica*, *E. murinus*, and the recently discovered *E. amsterdamensis* and *E. enhydrae* [2,3,4,5,6]. They represent class *Erysipelotrichia* in the phylum Bacillota (Firmicutes) [2,7]. *E. rhusiopathiae* strains grow on media supplemented with blood (whole or lysed), serum, or 0.1% Tween 80. They favor alkaline pH (optimum 7.2–7.6), usually produce H_2_S, and may be α-hemolytic [7]. Based on the structure of peptidoglycan antigens in *E. rhusiopathiae* strains, 15 serotypes are distinguished: 1a, 1b, 2, 4, 5, 6, 8, 9, 11, 12, 15, 16, 17, 19, and 21. Strains that do not cause the production of precipitating antibodies in immunized animals are classified as type N [2].

*E. rhusiopathiae* infections, both symptomatic and asymptomatic, are common in domestic pigs worldwide [8,9]. In Poland, however, erysipelas is also a serious problem in waterfowl farming [10]. Infection of birds takes the form of septicemia, leading to deaths in the flock. Anatomopathological examination shows hyperemia of internal organs, subcutaneous tissue, visceral fat, and muscles, as well as swelling of the liver, spleen, and air sacs [10,11]. Outdoor rearing is believed to increase birds’ exposure to *E. rhusiopathiae* bacteria, which can contaminate soil and water bodies. Prevention of erysipelas in waterfowl is limited due to the lack of vaccines for this group of animals and insufficient knowledge of predisposing factors for the disease [10]. Therefore, antibiotic therapy plays the most important role in combating *E. rhusiopathiae* infections in geese and ducks [12].

Currently available data on the antimicrobial susceptibility and genotypic resistance profiles of *E. rhusiopathiae* strains causing septicemia in waterfowl are very scarce [13,14]. Reports in this area, presenting the susceptibility of wild-type isolates to antimicrobial agents approved for the treatment of poultry diseases, are essential to developing effective infection control methods. Immediate implementation of appropriate therapy is crucial in the case of acute erysipelas, due to the risk of high mortality in the flock and thus huge economic losses. Moreover, monitoring the occurrence of antimicrobial resistant strains, including zoonotic ones, is an important task of the ‘European One Health Action Plan to combat antimicrobial resistance’ (launched in the EU in 2017, COM/2017/0339) [15].

The literature data show that *E. rhusiopathiae* strains can harbor various resistance genes that can be located on genomic islands or integrative and conjugative elements (ICEs), including the Tn916-like transposon. In single strains, the presence of prophage DNA-carrying resistance genes has also been demonstrated [14,16,17].

The aim of the study was to evaluate the antimicrobial susceptibility and determine the genotypic resistance profiles and serotypes of *E. rhusiopathiae* strains isolated from clinical cases of erysipelas in domestic geese and ducks reared in Poland, as well as to determine the potential mechanisms of resistance gene transfer occurring in these bacteria. An additional goal was to develop a multiplex PCR for detection of resistance genes and to determine the sequence type (ST) of multidrug-resistant (MDR) isolates. The comparative analyses included 260 *E. rhusiopathiae* strains whose whole genome sequences (WGSs) are publicly available.

## 2. Results

### 2.1. Identification of E. rhusiopathiae Isolates

Real-time PCR analysis confirmed that all 60 isolates obtained from geese and ducks with septicemia belonged to the species *E. rhusiopathiae*. In the case of four isolates, the affiliation to the species *E. rhusiopathiae* was additionally confirmed based on our previous analysis of the WGSs [14].

### 2.2. Serotyping

**Figure A1:**
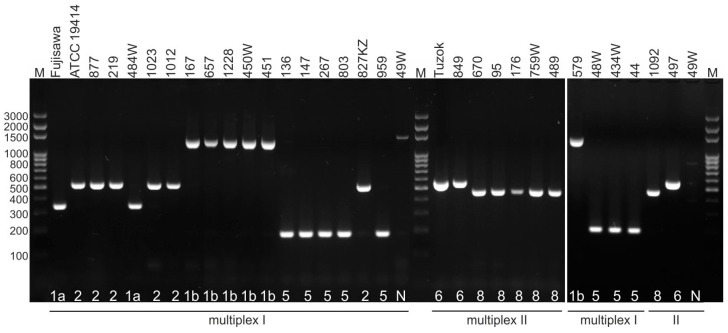
Serotypic differentiation of *E. rhusiopathiae* strains by multiplex PCR. PCR products were separated in 1.8% agarose. The labels below each lane correspond to serotypes. The strains Fujisawa serotype 1a, ATCC 19414 serotype 2, Tuzok serotype 6, and 267 serotype 5 were used as positive controls; M—molecular size marker.

The analyses performed showed that more than 38% of *E. rhusiopathiae* isolates belonged to serotype 5 (23/60; 38.3%) and 28.3% to serotype 1b (17/60). The remaining isolates represented serotypes 8 (9/60; 15.0%), 2 (6/60; 10.0%), and 6 (2/60; 3.3%). Two isolates for which no PCR product was obtained in any of the four multiplex PCRs were designated serotype N (2/60; 3.3%) (Table 1, Figure A1). Strains from ducks represented serotypes 5 (1/5), 1b (2/5), and 2 (2/5). The remaining serotypes were detected only in strains from geese. Interestingly, in the case of one sample obtained from a nine-week-old goose from the reproductive flock, two strains were isolated simultaneously, one of which belonged to serotype 8 (strain 818) and the other to serotype 5 (strain 155).

Among the 260 strains whose WGSs were retrieved from the GenBank database (https://www.ncbi.nlm.nih.gov/genbank/, accessed on 26 September 2024), serotypes 1a (93/260), 1b (57/260), and 2 (56/260) were dominant (Table 1). Serotype 8 was not identified in any strain. Among the 23 genomes of strains from poultry and wild birds, serovar 1b (8/23) and 5 (7/23) were most frequently identified. Serovar 6, like 5, was found only in isolates from birds (Table 1, Table S1).

### 2.3. Antimicrobial Susceptibility

All *E. rhusiopathiae* isolates (n = 60) were susceptible to β-lactam antibiotics, i.e., penicillin, ampicillin, and ceftiofur, and were also characterized by low MIC values of amoxicillin and amoxicillin + clavulanic acid (ca), i.e., ≤0.06 µg/mL. Low MICs in the range of 2–8 µg/mL were also observed for florfenicol. Most isolates showed resistance to tetracycline (85%) and enrofloxacin (80%). The percentages of strains resistant to other antimicrobial substances ranged from 3.3% (erythromycin) to 16.7% (lincosamides) (Table 2). Very high MICs (≥512 µg/mL) of gentamicin, neomycin and trimethoprim/sulfamethoxazole were obtained for all isolates except one, for which the MIC of gentamicin was 256 µg/mL. The MICs of streptomycin and spectinomycin ranged from 16 to ≥512 µg/mL (Table 2). Ten strains (16.7%) were multidrug resistant (MDR—strains with resistance to at least one antimicrobial substance from three or more groups). Interestingly, they most often represented serotype 8 (7/10), and less frequently serotype 5 (2/10) or serotype 2 (1/10) (Table A1).

Two erythromycin-resistant strains had concurrent tylosin resistance; surprisingly, one strain (1012, serotype 2) was resistant to tylosin but susceptible to erythromycin. Nine of 10 lincosamide-resistant strains were also resistant to tiamulin (Table A1). A correlation between tetracycline and enrofloxacin resistance was noted as well, except for three strains of serotype 2, which were resistant to tetracycline but susceptible to enrofloxacin (Table A1).

### 2.4. Genotypic Resistance Profiles

**Figure 1:**
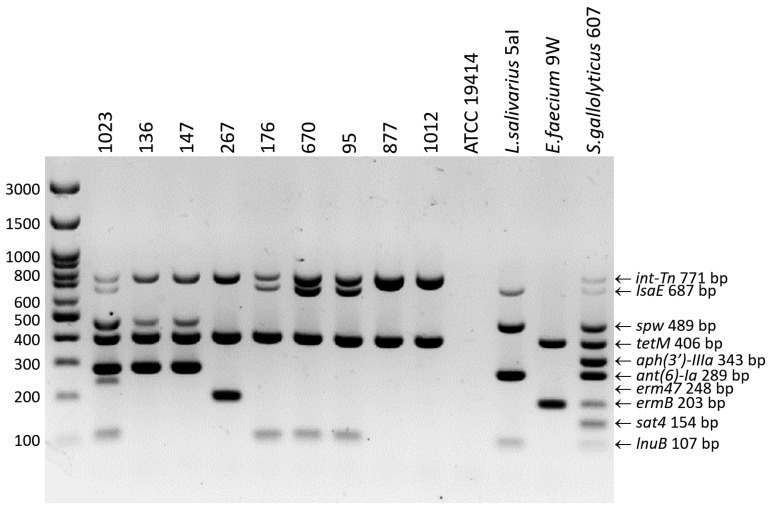
Electrophoretic separation of 10-plex PCR products in a 2% agarose gel. The strains *E. rhusiopathiae* 1023, 267, and 1012, *Ligilactobacillus salivarius* 5aI, *Enterococcus faecium* 9W, and *Streptococcus gallolyticus* 607 were used as positive controls. The negative control was the strain *E. rhusiopathiae* ATCC 19414.

The amplification patterns obtained using the 10-plex PCR developed in this study for the detection of *tetM*, *lsaE*, *lnuB*, *ant(6)-Ia*, *spw*, *aph(3′)-IIIa*, *sat4*, *ermB*, *erm47*, and *int-Tn* were fully consistent with the results of single PCRs and with the genotypic resistance profiles determined based on DNA sequence analysis of control strains. No nonspecific PCR products were visible on the gel. The results indicate that the new multiplex PCR protocol can be successfully used to detect resistance genes and transposons from the Tn916/Tn1545 family not only in *E. rhusiopathiae* strains but also in other Gram-positive bacteria, such as *Ligilactobacillus salivarius*, *Enterococcus faecium*, or *Streptococcus gallolyticus* (Figure 1).

The genotypic resistance profiles of *E. rhusiopathiae* strains were fully consistent with their phenotypic resistance. All tetracycline-resistant isolates contained the *tetM* gene (coding for ribosomal protection protein, which catalyzes the release of tetracycline from ribosomes in a GTP-dependent reaction). Resistance to macrolides, found in two isolates (no. 1023 and 267), whose WGSs were analyzed in our previous work [14], was caused by the *erm47* and *ermB* genes, respectively. All isolates resistant to tiamulin and lincosamides (n = 10) contained the *lsaE* gene (coding for ABC transporter) and the *lnuB* gene (coding for lincosamide nucleotide transferase). The exception was strain 267, whose resistance to lincosamides was determined by the *ermB* gene (coding for ribosomal methylase, which dimethylates a single adenine in 23S rRNA of 50S ribosomal subunit). In four strains characterized by high MICs of streptomycin (>512 µg/mL) and spectinomycin (256–>512 µg/mL), the presence of the *ant(6)-Ia* gene (coding for aminoglycoside nucleotidyltransferase) and the *spw* gene (coding for aminoglycoside nucleotidyltransferase of the ANT(9) family) was confirmed (Table A1). These genes have always coexisted, like *lnuB* and *lsaE* (Table A1, Figure 1).

Sequence analysis of the *gyrA* and *parC* genes showed that resistance of *E. rhusiopathiae* strains to enrofloxacin is due to a mutation at position 257 in the *gyrA* gene (C → T or C → A) and at position 242 in the *parC* gene (T → G). The mutations translate into the change Thr86 → Ile or Thr86 → Lys in the *gyrA* gene and Ser81 → Ile in the *parC* gene. Interestingly, a *gyrA*Thr86 → Lys mutation was recorded in all tested strains of serotype 5 and one strain of serotype 2, while the *gyrA*Thr86 → Ile mutation occurred in strains of serotypes 1b, 8 and 6 (Table 3).

### 2.5. Detection of ICE-Specific Genes and Prophage Regions

The *int-Tn* gene encoding the integrase of Tn916/Tn1545 transposons was detected in all *tetM*-positive isolates (Table A1, Figure 1). The *mobL* gene, encoding MobL relaxase, which is most likely responsible for DNA transfer by conjugation, was detected in several phenotypically resistant strains (n = 7) of serotypes 2 and 5. The *virB4* gene encoding the VirB4 protein specific for bacterial secretion system IV was found in only one strain, i.e., 1023, whose WGSs had been analyzed in our previous research [14] (Table A1).

**Figure 2:**
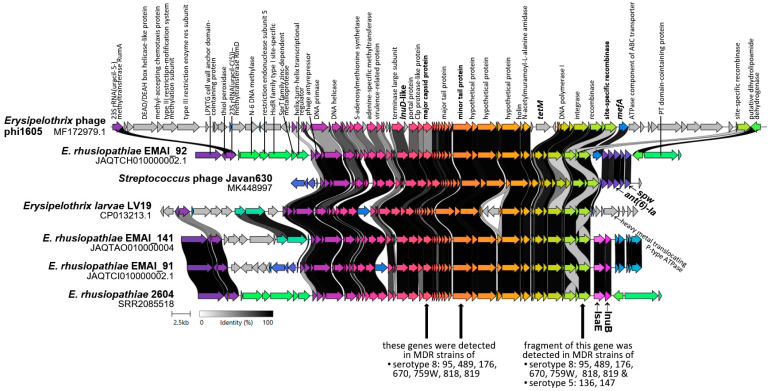
Clinker visualization showing homology between the *Erysipelothrix* phage phi1605 and *Streptococcus* phage Javan630 regions and genomes of *E. rhusiopathiae* (EMIAI_92, EMAI_141, 2604) and *E. larvae* LV19 strains. Arrows represent genes; the arrow’s colors represent the gene clusters identified by Clinker; homology between genes is represented by a gray gradient (%, the scale at the bottom of the figure).

BLAST analysis showed that regions homologous to the sequences of *Erysipelothrix* phage phi1605 (90,000 bp, GB: MF172979.1) [16] and *Streptococcus* phage Javan630 (48,058 bp, GB: MK448997.1) [18] are present in 18 of the 260 analyzed *E. rhusiopathiae* genomes (Table S1). Homologous prophage sequences were also confirmed in *E. larvae* strain LV19 and two strains of *Thomasclavelia ramosa* (DFI.6.112 and DFI.6.30, family *Erysipelotrichales*), as well as in several genomes of Gram-positive bacteria outside the *Erysipelotrichales* family, i.e., *Enterococcus*, *Streptococcus*, *Listeria*, *Clostridium*, and *Geosporobacter*. Interestingly, 17 out of 18 prophage-positive *E. rhusiopathiae* genomes contained the resistance genes: *tetM* (16/18), *lnuB* and *lsaE* (9/18), *ant(6)-Ia* and *spw* (6/18), *mph(B)* (3/18), *mef(A)* (2/18), *msr(D)* (2/18), *lnu(D)*-like, and *ermG* (1/18) (Table S1). Further analyses using BLAST and Clinker showed that most of these resistance genes are located within the prophage DNA (Table S1, Figure 2).

The results of analyses performed using publicly available WGSs of phages (Javan630 and phi1605) and *E. rhusiopathiae* strains suggested that also the MDR isolates examined in this study may harbor prophage regions containing resistance genes. Our assumptions were confirmed by PCR results, where the presence of three phage-specific genes, i.e., the gene coding for site-specific recombinase, minor tail protein, and major capsid protein, was detected in all MDR serotype 8 isolates (n = 7) (Figure 2). By amplifying a DNA region encompassing the phage site-specific recombinase gene and *lasE*, we showed that the *lsaE* gene is located within the prophage DNA in all of these isolates.

**Figure A2:**
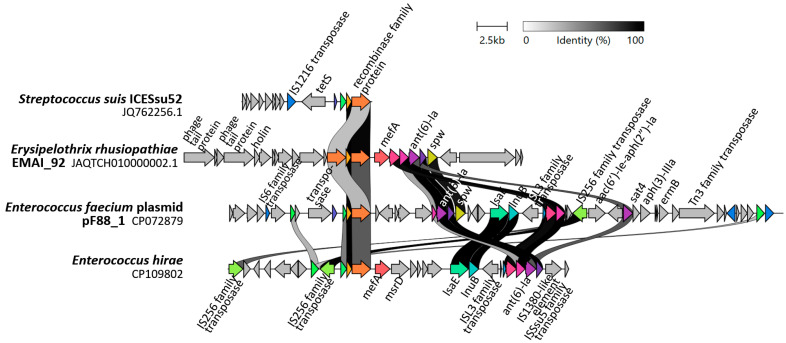
Clinker visualization of homology of bacterial DNA regions containing the gene encoding the recombinase family protein characteristic of *Streptococcus* phage Javan630 and *Erysipelothrix* phage phi1605. Arrows represent genes; the arrow’s colors represent the gene clusters identified by Clinker; homology between genes is represented by a gray gradient (%, the scale at the top of the figure).

In MDR isolate nos. 136 and 147 serotype 5, only the gene coding for phage site-specific recombinase was detected (Figure 2). Additional BLAST analysis showed that a 462-bp amplified region of this gene is present in many genomes of a wide variety of Gram-positive bacteria and may be located not only in the prophage DNA but also on a plasmid or an ICE, usually next to resistance genes (Figure A2).

**Figure 3:**
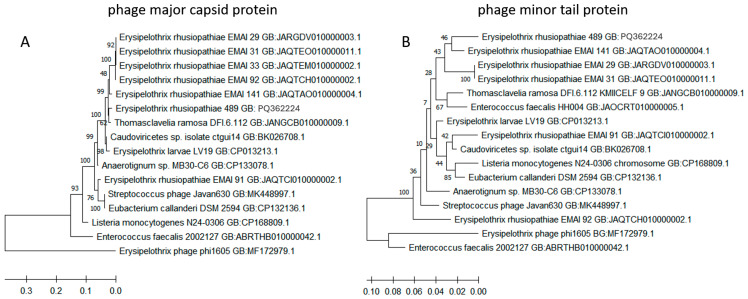
Dendrogram showing the similarity of the sequences of genes encoding the major capsid protein (**A**) and minor tail protein (**B**) detected in *E. rhusiopathiae* strain 489 to analogous sequences deposited in GenBank. Analysis was conducted by MEGA X software using the Maximum Likelihood method. The percentage of replicate trees in which the associated taxa were clustered together in the bootstrap test (500 replicates) is shown next to the branches. Scale bars show genetic distance.

BLAST analysis showed that the sequence of the gene encoding phage major capsid protein of the *E. rhusiopathiae* strain 489 serotype 8 (GB: PQ362224) is 98% homologous to the prophage region of *Thomasclavelia ramosa* strain DFI.6.112 (GB: JANGCB010000009.1) and the 93–97% homologous gene of *E. rhusiopathiae* strains EMAI 29, 31, 33, 91, 92, and 141 [19]. The similarity to the sequence of *Streptococcus* phage Javan630 was 93%, and to phage phi1605 (found in the *E. rhusiopathiae* ZJ strain in China)—only 67% (at 34% coverage) (Figure 3A). The sequence of the gene encoding phage minor tail protein in strain no. 489 serotype 8 (GB: PQ362225) was highly homologous to the analogous gene found in *E. rhusiopathiae* strains EMAI_141 (96%), EMAI_29, 31, 33, 92 (≥92%), and phage Javan630 (95%), and it was less similar (85%) to the sequences of phage phi1605 (Figure 3B). Sequences homologous to the gene coding of phage major capsid protein (≥85%) and minor tail protein (≥90%) of isolate no. 489 were also detected in the genomes of strains representing other genera of Gram-positive bacteria, i.e., *Enterococcus*, *Anaerotignum*, *Eubacterium*, and *Listeria* (Figure 3).

The results of the analysis using two phage genes allow us to conclude that the prophage regions present in MDR serotype 8 isolates are more similar to the sequence of prophage DNA detected in *E. rhusiopathiae* EMAI strains from Australia and *Streptococcus* phage Javan630 than to the *Erysipelothrix* phage phi1605.

### 2.6. MLST Results

Among the eight *E. rhusiopathiae* strains subjected to MLST analysis, the occurrence of two STs was demonstrated, i.e., 113 and 4. All serotype 8 strains, both multidrug-resistant (95, 489, 176, 759 W) and non-MDR (1092), represented ST 113. The same sequence type was also determined in MDR strain 8S serotype 8 from a pig in Poland. MDR strain 136 serotype 5 and non-MDR strain 451 serotype 1b represented ST 4, similarly to MDR strain 1023, whose WGSs were analyzed in our previous work [14]. It is worth noting that ST 4 and ST 113 differ only in the *galK* allele (Table 4).

**Figure 4:**
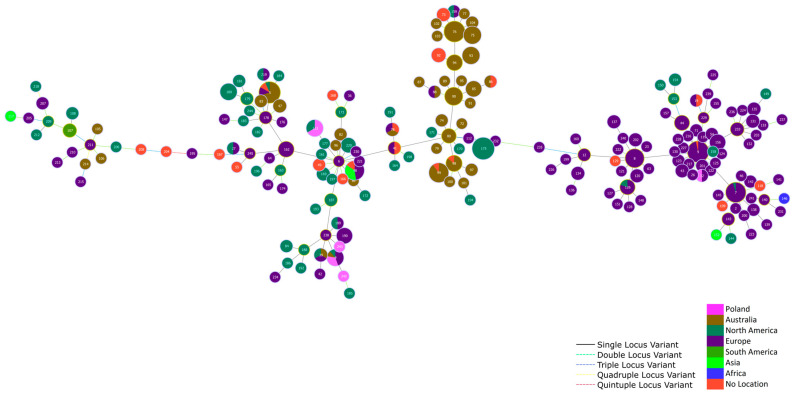
Minimum spanning tree based on the allelic profiles (*pta*, *galK*, *purA*, *ldhA*, *recA*, *gpsA*, and *prsA*) of 196 STs of *E. rhusiopathiae* strains (n = 557), including ST 4, 32, 113, 242, and 243 determined in isolates from waterfowl in Poland. Node size is proportional to the frequency of sequence occurrence.

Figure 4 shows the location of the studied *E. rhusiopathiae* strains representing ST 4, 113, 242, and 243 within a large group comprising isolates from different continents, i.e., North America, Europe, Australia, and Asia. In contrast, goose strain no. 584 representing ST32 was located in a large single-locus variant (SLV) group comprising mainly isolates from Europe (Figure 4).

## 3. Discussion

### 3.1. Serotypes of E. rhusiopathiae Strains

The high-to-moderate frequency of *E. rhusiopathiae* strains of serotypes 5 (38.3%), 1b (28.3%), 8 (15%), and 2 (10%) observed in waterfowl is largely consistent with our previous results on *E. rhusiopathiae* strains isolated from pigs in Poland [9]. High prevalence of strains of serotype 5 (53.3%) and 1b (43.3%) and low prevalence of serotype 2 (3.3%) have also been recorded in poultry (laying hens and turkeys) in Austria [20]. The same three serotypes (1b, 2, and 5) were identified by Bobrek and Gaweł [13] in *E. rhusiopathiae* isolates from domestic geese in Poland, with serotype 1b clearly predominating (55.3%). Different results were obtained in Germany, where among *E. rhusiopathiae* strains (n = 32) from birds and mammals, serotype 1a (62.5%) predominated, while strains of serotype 2 (28.1%) and N (9.4%) were isolated less frequently [21]. Clinical cases of swine erysipelas reported in Japan, China, and the UK are usually caused by strains of serotypes 1a, 1b, or 2 [22,23,24]. Serotyping is a basic method to determine the genetic diversity of isolates and can be helpful in epidemiological studies to assess the spread of strains in the environment. At the same time, it should be remembered that strains of the same serotype may differ significantly, e.g., in terms of resistance profile.

Analyses conducted in this paper indicate that strains of serotypes 5, 6, and 8 are characteristic of birds. However, in our previous studies we have shown the occurrence of *E. rhusiopathie* strains of these three serotypes also in pigs in Poland [9]. These observations, as well as the fact that serotype 1a is sporadically recorded in Poland, suggest that the prevalence of strains of a given serotype is to some extent correlated with the geographical region.

### 3.2. Antibiotic Susceptibility and Genotypic Resistance Profiles

The widespread susceptibility of *E. rhusiopathiae* strains to β-lactam antibiotics demonstrated in this study is fully consistent with several previous reports [9,25,26,27]. Low MICs of ampicillin and/or penicillin (≤0.25 µg/mL) were obtained for 100% of isolates obtained from pigs with symptoms of erysipelas in Poland [9], China [25], and Japan [26], as well as in *E. rhusiopathiae* strains isolated from various sources in Australia [27]. Contrasting results were presented by researchers from Austria, who tested strains of *E. rhusiopathiae* from poultry (mainly layer hens and turkeys) and observed ampicillin resistance (20% of strains, MIC ≥ 0.5 µg/mL) and penicillin resistance (40%, MIC ≥ 0.25 µg/mL) [20]. It should be noted, however, that those authors did not attempt to explain the mechanisms of this resistance, and to determine antimicrobial susceptibility they used MICRONAUT-S Lifestock/Equines GP microplates (MERLIN Diagnostika GmbH, Bornheim-Hersel, Germany), which in the case of *E. rhusiopathiae* strains can yield false positive results (based on our own observations, unpublished). This study is one of the first reports showing the susceptibility of *E. rhusiopathiae* bacteria to amoxicillin and amoxicillin + ca, and the obtained low MIC values ≤ 0.06 µg/mL indicate the lack of resistance of the tested strains to these antimicrobial agents. These results are very encouraging, as amoxicillin is the drug of choice for treating erysipelas in waterfowl [12,28]. The susceptibility of *E. rhusiopathiae* isolates from geese to amoxicillin and amoxicillin + cc (MIC ≤ 0.125 µg/mL) has also recently been demonstrated by Bobrek and Gaweł [13].

Infections in poultry are also treated with florfenicol, whose MIC for the *E. rhusiopathiae* strains studied here can be considered low (2–8 µg/mL). Similar susceptibility to chloramphenicols has been observed in *E. rhusiopathiae* strains isolated from pigs (florfenicol MIC range of 2–4 µg/mL) [9] and in *E. rhusiopathiae* strains from humans and various animals (other than poultry) collected in Australia (chloramphenicol MIC range of 8–16 µg/mL) [27].

The sporadic occurrence of *E. rhusiopathiae* isolates resistant to macrolides (2/60; 5%) observed in the present study is largely consistent with the findings of other authors [9,13,25,26]. Wholly different data were obtained in Austria, where as many as 76.7% of strains isolated from cases of erysipelas in poultry (chickens and turkeys) were classified as resistant to erythromycin [20]. High prevalence of macrolide resistance (53%) was also noted in a pool of isolates (obtained in 2012–2013) from pigs in China. The genes *ermT* and *ermA-like* were detected in most phenotypically resistant strains [29]. The erythromycin resistance recorded in the present study was determined by the presence of the *ermB* and *erm47* genes, and, as shown in our previous work, the *ermB* gene (in strain no. 267) was located within the prophage DNA, and the *erm47* gene (in strain no. 1023) was located on 138-kp ICE Er1023 [9]. While the gene *ermB* is common in Gram-positive bacteria, *erm47* has thus far only been confirmed in *Helcococcus kunzi* strain UCN99 from a patient with a diabetic foot ulcer in France [30] and in a strain of *Streptococcus suis* in Spain [31].

The prevalence of strains resistant to tiamulin (15.0%) is similar to that noted for *E. rhusiopathiae* isolates from pigs in China in 2015 (15.2%) [32]. The percentage of strains resistant to lincosamides (16.7%) was more than twice as high as in *E. rhusiopathiae* isolates from poultry in Austria (6.7%) [20] or from pigs in Poland (7.1%) [9]. Much higher prevalence of resistance to this group of antimicrobials (64–72%) was recorded in isolates from pigs in China [25,29]. The co-occurrence of the gene *lnuB* responsible for resistance to lincosamides with the gene *lsaE* determining resistance to pleuromutilins in the present study has also been confirmed by other authors [9,14,19]. The determinants *lnuB* and *lsaE* can be part of a resistance gene cluster, which may also include *ant(6)-Ia (aadE)*, *spw*, *ant(3′)-IIIa* (*aphA3*), and *sat4* genes [14,16]. Interestingly, as many as 7 of 10 strains found in our study to be resistant to lincomycin, clindamycin, and tiamulin belonged to serotype 8. In our earlier work, the lincomycin-clindamycin-tiamulin resistance profile was confirmed in one of 14 (7.1%) *E. rhusiopathiae* strains from pigs, and this was also a strain representing serotype 8 [9].

The very high percentage of *E. rhusiopathiae* strains resistant to tetracycline (85%) in the present study is consistent with the results of our previous research on *E. rhusiopathiae* strains from pigs (71.4% of isolates were resistant to tetracycline) [9]. Somewhat lower prevalence of resistance to this group of antibiotics has been described for *E. rhusiopathiae* strains isolated from domestic geese (in 2008–2018) in Poland (63.8%) [13] and from pigs in China and Japan (38–60%) [25,26,29,33]. The presence of the *tetM* gene in phenotypically resistant *E. rhusiopathiae* strains has also been confirmed by other authors [9,13,29].

The high prevalence of resistance to enrofloxacin in *E. rhusiopathiae* strains from waterfowl (80%) is consistent with results reported by Ding et al. [25] and Wu et al. [29], who confirmed resistance to fluoroquinolones (norfloxacin, levofloxacin, enrofloxacin, and ciprofloxacin) in ~70–91.7% of *E. rhusiopathiae* strains from pigs in China. A somewhat lower rate of resistance to enrofloxacin was noted among isolates from domestic geese in Poland (76.6%) [13] and from poultry in Austria (60%) [20]. The mechanism of resistance to fluoroquinolones confirmed in the present study (a mutation in the gene *gyrA* at position 257 and in *parC* at position 242) is in agreement with the findings of other authors [9,13,29]. It should be noted, however, that resistance to fluoroquinolones in these bacteria may also be due to a mutation at position 90 of gyrase subunit A [29]. The correlation observed in our study between the serotype and the type of mutation (in serotype 5 strains it was always Thr86 → Lys, while in isolates with serotypes 1b, 6, and 8 it was Thr86 → Ile86) is in agreement with the results of our previous research on *E. rhusiopathiae* isolates from pigs [9]. However, such a relationship was not shown in the study by Bobrek i Gaweł [13].

The correlation between the susceptibility of *E. rhusiopathiae* strains to tetracycline and enrofloxacin observed in the present study was also noted in isolates from pigs [9]. Due to the different molecular basis of resistance of *E. rhusiopathiae* to these tetracyclines, this correlation is difficult to explain.

The wide range of MIC values of streptomycin and spectinomycin (16–>512 µg/mL) noted in this study is largely consistent with data presented by Coutinho et al. [34], who showed that the MICs of spectinomycin in *E. rhusiopathiae* strains from pigs ranged from 8 to >64, with an MIC_90_ of 32 µg/mL. The authors suggested that *E. rhusiopathiae* should be considered spectinomycin-resistant at MIC ≥128 µg/mL. The results of our study, however, indicate that acquired resistance to streptomycin and spectinomycin determined by the presence of the genes *ant(6)-Ia* and *spw*, respectively, is correlated with higher MICs of these antibiotics, from 256 to >512 µg/mL (in the only strain with a spectinomycin MIC of 128 µg/mL, *spw* was not detected). Therefore, an MIC of 256 µg/mL should be considered as a possible cut-off point for spectinomycin. The same MIC value is a potential cut-off point for streptomycin (MICs of *ant(6)-Ia*-negative isolates ranged from 16 to 128 µg/mL). However, the validity of these suggestions requires further research on a larger number of *E. rhusiopathiae* strains. The presence of the determinant *ant(6)-Ia* encoding aminoglycoside nucleotidyltransferase has previously been confirmed in multidrug-resistant *E. rhusiopathiae* strains from pigs (at streptomycin MIC = 512 µg/mL) [32,34].

The high MICs noted in this study for gentamicin, neomycin, and trimethoprim/sulfamethoxazole (256–≥512 µg/mL) obtained for all *E. rhusiopathiae* isolates indicate the occurrence of natural resistance in these bacteria. The lack of sensitivity of *E. rhusiopathiae* to neomycin and kanamycin was first described by Wood [35], who developed a medium with these antibiotics for isolating *E. rhusiopathiae* strains (at that time *Erysipelothrix insidiosa*). Natural resistance of *E. rhusiopathiae* bacteria to aminoglycosides (neomycin, kanamycin, amikacin, and gentamicin) and folic acid inhibitors (sulfadiazine and trimethoprim) has been confirmed by several other authors [9,25,34,36].

### 3.3. Potential Mechanisms of Resistance Gene Transfer

Mobile genetic elements (MGEs), i.e., plasmids, integrative and conjugative elements (ICEs), and bacteriophages, are crucial for horizontal gene transfer (HGT) and the spread of antibiotic resistance in bacteria. Studies conducted so far show that in *E. rhusiopathiae* bacteria, transfer of resistance genes may be determined by ICEs or bacteriophages [14,16,17].

The occurrence of prophage DNA (Javan 630-like) associated with resistance genes (*lsaE* and most likely *lnuB*) in MDR isolates of serotype 8 demonstrated in this work is consistent with the studies by Gu et al. [16], who confirmed the presence of temperate phage phi1605 carrying the *mef(A)*, *mrs(D)*, *lnu(D)*-like, and *tetM* genes in the *E. rhusiopathiae* ZJ strain. The ability of this phage to infect recipient *E. rhusiopathiae* strain in the presence of mitomycin C and to transfer resistance genes was also demonstrated [16]. The results of our analyses showing that the *lsaE* and *lnuB* as well as *ant(6)-Ia* and *spw* determinants in most *E. rhusiopathiae* strains are located within the prophage DNA (Table S1) indicate that bacteriophages play a key role in the horizontal transfer of these resistance genes. The fact that prophage DNA was found only in serotype 8 isolates may be related to the presence of serotype-dependent phage receptors. In previous work, prophage regions linked to the *ermB* gene were detected in one of the *E. rhusiopathiae* isolates tested (no. 260), but they did not show homology to any of the three phage genes detected in serotype 8 isolates or to the Javan630 and phi1605 phage sequences.

The coexistence of the *tetM* gene and *int-Tn* gene (Tn916 transposon integrase gene) noted in this study is consistent with earlier analyses, which showed that the *tetM* gene in *E. rhusiopathiae* is always located within 18 kp of the Tn916-like conjugative transposon [14]. *E. rusiopathiae* strains may contain larger ICEs containing resistance genes [14,16]. In the previous studies, in one of the isolates from waterfowl, i.e., 1023, we demonstrated the presence of ICEEr1023 (130 kb) carrying the *ant(6)-Ia–spw–lsa(E)–lnu(B)* cluster and the *erm47* and *tetM* genes. In another strain from geese, i.e., 1012, we confirmed the presence of ICEEr1012 (74 kb) containing the *tetM* gene. Within both ICEs, a Tn916-like transposon and the *mobL* gene were present. The latter encodes the MobL relaxase, which initiates bacterial conjugation through a site- and strand-specific nick in the oriT region of the conjugation element. Finally, a single-stranded DNA that is transferred from the donor to the recipient cell via the multicomponent protein pore called the type IV secretion system (T4SS) [37].

The presence of the *mobL* gene confirmed in these studies also in several other isolates (i.e., 877, 86, 13, 815, 136, 147) makes it highly probable that their genomes contain ICE homologous to ICEEr1012. Interestingly, these were only isolates of serotypes 5 and 2, including 3 MDR strains. The presence of ICEs or genomic islands carrying resistance genes was also demonstrated in *E. rhusiopathiae* strains from pigs in China [16,17]. In resistant isolates no. 136 and 147 of serotype 5, the presence of ICE is additionally indicated by the site-specific recombinase homologous to the integrase of phages Javan630 and phi1605 phage recombinase gene. ICE-encoded recombinases, which enable both integration and excision, are often homologous to phage integrases, and, like temperate phages, many ICEs insert at a specific attachment site in the bacterial chromosome (attB) [37].

### 3.4. Sequence Types of E. rhusiopathiae Isolates

The results of MLST analysis performed on selected *E. rhusiopathiae* isolates from waterfowl were compared to STs of 557 strains whose sequences are publicly available. The ST 113 assigned to six MDR serotype 8 isolates from Poland was also determined in 3 strains from pigs from Canada, which belonged to the serotype 1b. ST 4 identified in isolates no. 136, 1023, and 451 (serotype 5 and 1b) was also recorded in several *E. rhusiopathiae* strains from domestic and wild pigs from different countries, including Italy (3 strains of serotype 1b), Denmark (1 strain of serotype 2), Australia (1 strain of serotype 21), and Canada (1 strain of serotype 1b) (Table S2). The ST in *E. rhusiopathiae* strains therefore does not appear to be correlated with either the host species or the serotype.

## 4. Materials and Methods

### 4.1. Isolation of E. rhusiopathiae Strains

The research material comprised 60 *E. rhusiopathiae* isolates from domestic geese (n = 54) and ducks (n = 5) from 59 farms located in Poland, mainly in the Wielkopolskie Voivodeship. Dead birds were delivered to the laboratory for diagnostic purposes during the period from 2019 to 2021. *E. rhusiopathiae* bacteria were isolated from the internal organs (heart, spleen, and air sacs) of dead domestic geese and ducks. Before taking the smear, the organs were incised with a sterile scalpel. Swabs were inoculated onto Columbia agar with 5% sheep blood (GRASO Biotech, Owidz, Poland), and the plates were incubated for up to 48 h [10]. Small (up to 1 mm), flat colonies, some of which were hemolytic, were propagated on BHI broth with the addition of 0.1% Tween 80 (Merck, Warsaw, Poland) to obtain pure cultures. Broth suspensions of bacteria supplemented with 20% glycerol were stored at −80 °C.

### 4.2. Identification of E. rhusiopathiae Strains

Isolates were identified using the EXOone *Erysipelothrix rhusiopathiae* test (Exopol, Spain) based on the real-time PCR technique. Amplification was carried out in the Applied Biosystems^®^ 7500 FAST Real-Time PCR System with High Resolution Melt software v3.0.1 (Thermo Fisher Scientific, Waltham, MA, USA) according to the manufacturer’s protocol, i.e., 95 °C—5 min; 40 cycles of 95 °C—15 s, 60 °C—1 min. The template included with the kit and DNA of *E. rhusiopathiae* strain ATCC 19414 were used as positive controls. A PCR product was detected in the FAM channel and the internal control (IC) in the HEX channel, in accordance with the kit manufacturer’s guidelines [10].

### 4.3. Serotyping of E. rhusiopathiae Strains

Serotyping was based on four multiplex PCR protocols [2,38]. The primer sequences, annealing temperature, and size of PCR products are listed in Table S3. PCR reactions were performed using DreamTaq Green DNA Polymerase (Thermo Fisher Scientific Baltics UAB, Vilnius, Lithuania). The following *E. rhusiopathiae* strains were used as positive controls: Fujisawa serotype 1a (GB: AP012027.1), ATCC 19414 (NCTC 8163) serotype 2 (GB: NZ_LR134439.1), Tuzok serotype 6 (GB: SRR11123328), Bano serotype 21, and wild-type strain 267, in the genome of which we confirmed in silico the presence of sequences determining serotype 5 (ENA: ERR12736634). Strain ATCC 19414 was obtained from Argenta (Poznań, Poland), and other reference strains were kindly provided in the form of genomic DNA by Dr. Yoshihiro Shimoji, National Institute of Animal Health, Japan.

The comparative analysis included 260 *E. rhusiopathiae* strains whose WGSs are publicly available (Table S1). The serotype of these strains was determined in silico using the serotype-determining sequences deposited in the GenBank database [2] and the BLAST tool (https://blast.ncbi.nlm.nih.gov/Blast.cgi, accessed on 17 September 2024).

### 4.4. Antimicrobial Susceptibility Testing (AST)

The antimicrobial susceptibility of *E. rhusiopathiae* isolates was determined by the broth microdilution method, as previously described [9]. The following antimicrobial agents were used in the AST: penicillin, ampicillin, amoxicillin, amoxicillin + ca (5:1), ceftiofur, tetracycline, erythromycin, tylosin, clindamycin, lincomycin, tiamulin, enrofloxacin, streptomycin, spectinomycin, gentamicin, neomycin, trimethoprim + sulfamethoxazole (1:19), and florfenicol. All antimicrobial agent powders were obtained from Merck (Warsaw, Poland). Ready-to-use solutions of tiamulin (Biomutin, 200 mg/mL) and spectinomycin (100 mg/mL) were purchased from BIOWET DRWALEW S.A. (Drwalew, Poland) and Merck (Warsaw, Poland), respectively. The drug Taromentin (powder for solution for infusion) (Polfa Tarchomin, Warsaw, Poland) was used as a source of amoxicillin + ca.

Antibiotic/chemotherapeutic stock solutions were prepared by dissolving the powder in appropriate solvents (according to the recommendations of CLSI or the manufacturer). The AST was performed in BHI broth (BTL, Łódź, Poland) containing 0.1% Tween 80, as previously described [9]. Plates were incubated at 37 °C in 5% CO_2_ for 24–26 h, and MIC values were read as the lowest concentration of an antimicrobial agent at which visible growth was inhibited. Quality control of antimicrobial agents was carried out using *E. coli* strain ATCC 25922 and Müller-Hinton broth [39].

Categorization of *E. rhusiopathiae* strains as susceptible, intermediate resistant, and resistant was carried out based on CLSI guidelines (document Vet06, 2017) [40]. In the case of tetracycline, tylosin, lincomycin, and tiamulin, the recommendations of Dec et al. [9] were used. For amoxicillin and amoxicillin + ca, the CLSI cut-off points recommended for ampicillin were adopted. The categorization did not include florfenicol, trimethoprim/sulfamethoxazole, or aminoglycoside antibiotics due to the lack of available guidelines and unimodal MIC distribution (Table 5).

### 4.5. Detection of Resistance Genes and Development of 10-Plex PCR

Conventional PCR was used to detect the presence of genes that confer resistance to aminoglycosides (*aac(6′)-Ie-aph(2”)-Ia*, *aph(3′)-IIIa*, *ant(4′)-Ia*, *aph(2”)-Ib*, *aph(2”)-Ic*, *aph(2”)-Id*, *ant(6)-Ia*, and *ant(9)-Ia*), spectinomycin (*spw*), tetracyclines (*tetK*, *tetL*, *tetM*, and *tetO*), pleurumutilins (*lsaE*), macrolides and/or lincosamides (*ermA, ermB*, *ermT*, and *mefA/E, lnuB*), and streptothricin (*sat4*). Primer sequences, PCR product size, and annealing temperature are shown in Table S4.

The primers for amplification of *lnuB*, *ant(6)-Ia*, *ermB*, *erm47*, *spw*, *sat4*, *aph(3′)-IIIa*, and *int-Tn* were designed using the Primer-BLAST tool (https://www.ncbi.nlm.nih.gov/tools/primer-blast/, accessed on 30 June 2024).

In order to develop a multiplex PCR protocol for detection of the *lnuB*, *ant(6)-Ia*, *ermB*, *erm47*, *tetM*, *lsaE*, *spw*, *aph(3′)-IIIa*, *sat4*, and *int-Tn* genes, the interactions between the primers, i.e., their ability to form primer dimers and self-dimers, were assessed using the Multiple Primer Analyzer (https://www.thermofisher.com, accessed on 2 July 2024). First, the primers were used in single PCRs to assess their amplification efficiency in the annealing temperature range of 55–60 °C and the possible formation of non-specific PCR products. Analyses were performed on control strains (Table 6).

Amplification was performed using DreamTaq Green DNA polymerase (Thermo Fisher Scientific Baltics UAB, Vilniaus, Lithuania), and the final composition of the reaction mixture for 10-plex PCR was as follows: 10× DreamTaq Green Buffer—1.25 µL, dNTP mix (10 mM)—0.75 µL, each primer (10 pmol/µL)—0.23 µL, template DNA (15–30 ng/µL)—0.7 µL, DreamTaq DNA polymerase (5 U/µL)—0.07 µL, and nuclease-free water—up to 12.5 µL. DNA amplification was performed under the following conditions: initial denaturation for 5 min at 94 °C, 30 cycles of 40 s at 94 °C (denaturation), 40 s at 57.3 °C (annealing), 1 min at 72 °C (extension), and 8 min at 72 °C (final extension).

### 4.6. Sequence Analysis of the gyrA and parC Genes

To determine the mechanism of resistance of *E. rhusiopathiae* strains to quinolones, the *gyrA* and *parC* genes of representative enrofloxacin-susceptible and enrofloxacin-resistant strains were amplified using the primers listed in Table S4 and sequenced using the Sanger method. Amino acid (aa) sequences were predicted using the NCBI translate tool ORF finder (https://www.ncbi.nlm.nih.gov/orffinder/, accessed on 12 March 2024). The *gyrA* sequence of reference strain *E. rhusiopathiae* ATCC 19414 (enrofloxacin-susceptible) was retrieved from the NCBI GenBank database (GB: LR134439.1).

### 4.7. Detection of ICE-Specific Genes and Prophage DNA

Genes specific for ICEs occurring in *E. rhusiopathiae* (ICEEr010, ICEEr1012, ICEEr1023, Tn916-like) [14,16] were detected by PCR using primers listed in Table S5. These were the *int-Tn* gene encoding the Tn916/Tn1545 transposon integrase, the *virB4* gene coding for type IV secretory pathway protein VirB4, and the *mobL* gene coding for MobL relaxase involved in the conjugative transfer of DNA.

Prophage regions were detected by PCR using primers specific for selected phage genes present in the genome of phage Javan630 (GB: MK448997.1) [18] and phage phi1605 (GB: MF172979.1) [16]. These were gene-encoding site-specific recombinase, major capsid protein, and minor tail protein (Table S5). Primers for detection of the sequence comprising the phage recombinase gene and the *lsaE* resistance gene were designed on the template of the *E. rhusiopathiae* strain EMAI_141 (GB: JAQTAO010000004.1) [19]. Comparative analysis of phage sequences was performed using BLAST (https://www.ncbi.nlm.nih.gov/tools/primer-blast/, accessed on 13 September 2024), Clinker (https://cagecat.bioinformatics.nl, accessed on 21 September 2024) [43], and MEGA X software (https://www.megasoftware.net/, accessed on 26 November 2022) [44].

The comparative analyses (detection of ICE-specific genes and prophage DNA, analysis of the occurrence of resistance genes within phage DNA) included 260 *E. rhusiopathiae* strains whose WGSs are publicly available (Table S1).

### 4.8. Multilocus Sequence Typing

The MLST of *E. rhusiopathiae* strains was performed for 10 isolates, including 7 MDR ones. Amplification of the *pta, galK, purA, ldhA, recA, gpsA*, and *prsA* genes [21] was performed using primers listed in Table S6. The allele sequences were screened against the MLST scheme updated by Webster et al. [19] using mlst v.2.19.0 (https://github.com/tseemann/mlst, accessed on 16 September 2024). A minimum spanning tree based on MLST allele numbers was computed in PHYLOViZ Online (https://online.phyloviz.net/index, accessed on 16 September 2024) with the goeBURST full MST function. The analysis included a total of 557 *E. rhusiopathiae* strains (Table S2).

## 5. Conclusions

This study provides new data on the serotypes and antimicrobial susceptibility of *E. rhusiopathiae* strains causing erysipelas in geese and ducks in Poland. For the first time, the occurrence of *E. rhusiopathiae* isolates representing serotype 8 was reported in poultry. Of particular importance are the findings regarding the susceptibility of *E. rhusiopathiae* isolates to antimicrobials approved for the treatment of poultry diseases. These data may contribute to expanding the current CLSI recommendations with guidelines that would allow veterinary diagnosticians worldwide to uniformly categorize *E. rhusiopathiae* strains as susceptible or resistant to antimicrobial agents used in poultry farming.

The results of our research indicate that β-lactam antibiotics should be the first choice for the treatment of erysipelas in waterfowl, while aminoglycosides, folate inhibitors, tetracyclines, and fluoroquinolones should not be considered. The low MIC values of florfenicol also suggest that it may be effective in the treatment of *E. rhusiopathiae* infections, but due to its broad spectrum of activity, it should be used only when treatment with narrow-spectrum agents is not possible. As *E. rhusiopathiae* is a zoonotic microorganism, the data obtained on antimicrobial susceptibility may also be helpful in the treatment of erysipelas in humans.

The presence of prophage DNA and ICE-specific genes, including the Tn916/Tn1545 transposon integrase gene, in the genomes of the isolates tested indicates that bacteriophages and ICEs play a key role in the spread of resistance genes in *E. rhusiopathiae*. The MDR strains of serotype 8 representing ST 113 seem to be specific to the area of Poland.

Further studies based on WGS analysis will allow us to determine the sequence of prophage regions occurring in these strains and to determine the genetic environment of all resistance genes detected in MDR strain nos. 136 and 147 serotype 5.

The multiplex PCR developed for the detection of 9 resistance genes and the *int-Tn* gene encoding the integrase of transposons from the Tn916/Tn1545 family makes it possible to significantly reduce both the time and cost of analysis compared to single PCR. Moreover, it can be used to detect resistance genes not only in *E. rhusiopathiae* but also in other Gram-positive bacteria.

## Figures and Tables

**Table 1:** Number and percentage of *E. rhusiopathiae* strains representing individual serotypes.

Serotype	*E. rhusiopathiae* Strains Tested in This Work (n = 60)	*E. rhusiopathiae* Strains Whose WGS Were Derived from GenBank (n = 260)	Notes
1a	1 [1.7%]	92 [35.4%]	
1b	17 [28.3%]	57 [21.9%]	
2	6 [10.0%]	56 [21.5%]	
1a/2	0	22 [8.5%]	
1a/1b	0	1 [0.4%]	
2/15	0	1 [0.4%]	
5	23 [38.3%]	7 [2.7%]	all strains from birds *
6	2 [3.3%]	2 [0.8%]	all strains from birds *
8	9 [15.0%]	0	all strains from birds *
9	0	3 [1.1%]	
11	0	1 [0.4%]	
15	0	1 [0.4%]	
16	0	1 [0.4%]	
17	0	2 [0.8%]	
19	0	1 [0.4%]	
21	0	11 [4.2%]	
23	0	1 [0.4%]	
N	2 [3.3%]	1 [0.4%]	

* Strains whose WGSs were analyzed originated from both poultry and wild birds; detailed information on the bird species is provided in Table S1.

**Table 2:** Distribution of MIC values obtained in antimicrobial susceptibility tests for *E. rhusiopathiae* isolates (n = 60) and resistance genes detected.

	≤0.06	≤0.125	≤0.25	0.5	1	2	4	8	≥16	32	≥64	128	256	≥512	Number (%) of Resistant Isolates
PEN	60														0
AMP	37	22	1												0
AMX	60														0
AMC	60														0
CEF	59		1												0
TET			1	2	6				2*^tetM^*	22*^tetM^*	27*^tetM^*				51 (85%)
ENR		12						29	19						48 (80%)
ERY	2	34	21	1						1*^erm47^*	1*^ermB^*				2 (3.3%)
TYL	2	49	5	1	1*^erm47^*	1					1*^ermB^*				3 (5.0%)
CLI	42	7	1		2*^lnuB^*	5*^lnuB^*	2*^lnuB^*		1						10 (16.7%)
LIN			15	24	8	1	1	1			10*^lnuB^*^(9)^ *^ermB^* ^(1)^				10 (16.7%)
TIA				4	10	22	12	3			9*^lsaE^*				9 (15%)
FLO						3	50	7							NA
STR									2	9	23	22		4*^ant(6)-Ia^* *^spw^*	NA
SPE									9	28	18	1	2*^ant(6)-Ia, spw^*	2*^ant(6)-Ia,^* *^spw^*	NA
GEN													1	59	NA
NEO														60	NA
TR/S														60	NA

MIC values highlighted in grey indicate resistance, and those highlighted in blue indicate medium susceptibility. The number of strains in which the gene was detected is given in brackets next to the gene name. Where there is no numerical value after the gene name, the gene was present in all isolates with a given MIC value. Legend: PEN—penicillin; AMP—ampicillin; AMX—amoxicillin; AMC—amoxicillin + ca; CEF—ceftiofur; TET—tetracycline; ENR—enrofloxacin; ERY—erythromycin; TYL—tylosin; CLI—clindamycin; LIN—lincomycin; TIA—tiamulin; FLO—florfenicol; STR—streptomycin; SPE—spectinomycin; GEN—gentamicin; NEO—neomycin; TR/S—trimethoprim/sulfamethoxazole; NA—not applicable.

**Table 3:** Sequence analysis of the quinolone resistance-determining region (QRDR) in the *gyrA* and *parC* genes in enrofloxacin-susceptible and resistant *E. rhusiopathiae* strains.

Isolate	Serotype	Enrofloxacin MIC [µg/mL]		Mutation at Position 257 of the *gyrA* Gene	GB or ENA Acc. No.	Mutation at Position 242 of the *parC* Gene	GB or ENA Acc. No.
ATCC 19414	2	≤0.25	S	Thr_86_ (ACA)	LR134439.1	Ser_81_ (AGT)	LR134439.1
1012	2	≤0.125	S	Thr_86_ (ACA)	OP921306	Ser_81_ (AGT)	ERR12736636
1023	2	16	R	Ile_86_ (ATA)	OP921307	Ile_81_ (ATT)	ERR12736637
827KZ	2	8	R	Lys_86_ (AAA)	PQ015304	Ile_81_ (ATT)	PQ015313
584	5	≤0.125	S	Thr_86_ (ACA)	ERR1273663	Ser_81_ (AGT)	ERR1273663
267	5	8	R	Lys_86_ (AAA)	OQ625325	Ile_81_ (ATT)	ERR12736634
136	5	8	R	Lys_86_ (AAA)	PQ015305	Ile_81_ (ATT)	PQ015314
434W	5	8	R	Lys_86_ (AAA)	PQ015306	Ile_81_ (ATT)	PQ015315
95	8	16	R	Ile_86_ (ATA)	OP921308	Ile_81_ (ATT)	PQ015321
759W	8	8	R	Ile_86_ (ATA)	PQ015307	Ile_81_ (ATT)	PQ015318
1092	8	16	R	Ile_86_ (ATA)	PQ015308	Ile_81_ (ATT)	PQ015319
849	6	≤0.125	S	Thr_86_ (ACA)	PQ015309	Ser_81_ (AGT)	PQ015322
497	6	16	R	Ile_86_ (ATA)	PQ015310	Ile_81_ (ATT)	PQ015320
657	1b	≤0.125	S	Thr_86_ (ACA)	OP921309	Ser_81_ (AGT)	PQ015323
167	1b	16	R	Ile_86_ (ATA)	PQ015311	Ile_81_ (ATT)	PQ015317
526	1b	16	R	Ile_86_ (ATA)	PQ015312	Ile_81_ (ATT)	PQ015316

Legend: S—susceptible, R—resistant; GB—GenBank; ENA—European Nucleotide Archive.

**Table 4:** MLST analysis results.

Isolate	Serotype	Host	Year of Isolation	Resistance Genes	ICE- and Phage-Specific Genes	*gpsA*	*recA*	*purA*	*pta*	*prsA*	*galK*	*ldhA*	ST
95	8	goose	2020	*tetM*, *lnuB*, *lsaE*	*int-Tn*, rec ^1^, mcp ^2^, mtp ^3^	2	4	2	2	2	6	2	113
759W	8	goose	2019	*tetM*, *lnuB*, *lsaE*	*int-Tn*, rec, mcp, mtp	2	4	2	2	2	6	2	113
176	8	goose	2020	*tetM*, *lnuB*, *lsaE*	*int-Tn*, rec, mcp, mtp	2	4	2	2	2	6	2	113
489	8	goose	2020	*tetM*, *lnuB*, *lsaE*	*int-Tn*, rec, mcp, mtp	2	4	2	2	2	6	2	113
8S	8	pig	2019	*tetM*, *lnuB*, *lsaE*	*int-Tn*, rec, mcp, mtp	2	4	2	2	2	6	2	113
1092	8	goose	2021	*tetM*	*int-Tn*	2	4	2	2	2	6	2	113
451	1b	duck	2021	*tetM*	*int-Tn*	2	4	2	2	2	5	2	4
136	5	goose	2020	*tetM*, *ant(6)-Ia*, *spw*	*int-Tn*, *mobL*, rec	2	4	2	2	2	5	2	4
1023 *	2	goose	2021	*tetM*, *lnuB*, *lsaE*, *spw*, *ant(6)-Ia*, *erm47*	*int-Tn*, *mobL*, *virB4*	2	4	2	2	2	5	2	4
1012 *	2	goose	2020	*tetM*	*int-Tn*	2	4	2	2	4	5	2	242
267 *	5	goose	2021	*tetM*, *ermB*	*int-Tn*, prophage detected ^4^	2	24	2	2	2	5	2	243
584 *	5	goose	2021	none	none	5	1	1	1	1	1	1	32

*—the ST of this strain was determined in our previous paper based on WGS analysis [14]. ^1^—gene coding for recombinase family protein (phage integrase). ^2^—gene coding for phage major capsid protein. ^3^—gene coding for phage minor tail protein. ^4^—analysis of prophage DNA sequence was presented in our previous paper [14]; it shows no homology to phage Javan630 or phi1605, but contains the *ermB* gene.

**Table 5:** MIC (µg/mL) breakpoints used to categorize *E. rhusiopathiae* strains as susceptible (S), intermediate (I), and resistant (R) in the broth microdilution method.

Antimicrobial Agent	Breakpoints			Reference
	S	I	R	
Penicillin	≤0.12	–	–	CLSI VET06 [40]
Ampicillin	≤0.25	–	–	CLSI VET06 [40]
Amoxicillin	–	–	–	No recommendations
Amoxicillin + ca	–	–	–	No recommendations
Ceftiofur	≤2	4	≥8	CLSI VET06 [40]
Erythromycin	≤0.25	0.5	≥1	CLSI VET06 [40]
Tylosin	≤0.25	0.5	≥1	Dec et al. [9]
Clindamycin	≤0.25	0.5	≥1	CLSI VET06 [40]
Lincomycin	≤2	4 -8	≥16	Dec et al. [9]
Tiamulin	≤16	–	≥32	Dec et al. [9]
Enrofloxacin	≤0.5	1	≥2	CLSI VET06 [40]
Tetracycline	≤4	8	≥16	Dec et al. [9]
Florfenicol	–	–	–	No recommendations
Gentamicin	–	–	–	No recommendations
Neomycin	–	–	–	No recommendations
Streptomycin	–	–	–	No recommendations
Spectinomycin	–	–	–	No recommendations
Trimethoprim/ sulfamethoxazole	–	–	–	No recommendations

**Table 6:** Bacterial strains used as controls in 10-plex PCR.

	Genotypic Resistance Profile	Acc. No.	Ref.
*E. rhusiopathiae* 1023	*tetM*, *lsaE*, *lnuB*, *spw*, *ant(6)-Ia*, *erm47; int-Tn*	ENA: ERR12736637	[14]
*E. rhusiopathiae* 267	*tetM*, *ermB; int-Tn*	ENA: ERR12736634	[14]
*E. rhusiopathiae* 1012	*tetM; int-Tn*	ENA: ERR12736636	[14]
*E. rhusiopathiae* ATCC 19414	none	GB: NZ_LR134439.1	unpublished
*Streptococcus gallolyticus* 607	*tetM*, *lsaE*, *lnuB*, *spw*, *ant(6)-Ia*, *sat4 aph(3′)-IIIa; int-Tn*,	Not applicable	unpublished
*Enterococcus faecium* 9W	*tetM*, *ermB*	Not applicable	[41]
*Ligilactobacillus salivarius* 5aI	*ant(6)-Ia*, *spw*, *lsaE*, *lnuB*	GB: MK091478.1; MK091477.1	[42]

GB—GenBank; ENA—European Nucleotide Archive.

## Data Availability

The nucleotide sequences reported in this paper have been deposited in the NCBI GenBank database under the following accession numbers: OP921301-OP921305, PQ015304-PQ015312 (*gyrA*), PQ015313-PQ015323 (*parC*), PQ362224 (gene coding for major capsid protein), and PQ362225 (gene encoding phage minor tail protein).

## Supplementary Materials

The following supporting information can be downloaded at https://www.mdpi.com/article/10.3390/ijms252212192/s1 [45,46,47,48,49,50,51,52,53,54,55,56,57,58,59].

## Appendix A

**Table A1:** Detailed results of the antimicrobial susceptibility test and detection of resistance genes and ICEs-specific genes.

	Isolate	Ser.	AMP	PEN	AMX	AMC	CEF	ERY	TYL	LIN	CLI	TIA	TET	ENR	STR	SPE	GEN	NEO	TR/S	FLO	Resistance Genes	ICE-Specific Genes	Prophage Encoded Genes
1	484W	1a	≤0.06	≤0.06	≤0.06	≤0.06	≤0.06	0.125	0.125	1	≤0.06	4	≤0.25	≤0.125	128	32	>512	>512	>512	4			
2	219	2	≤0.06	≤0.06	≤0.06	≤0.06	≤0.06	0.125	0.125	0.25	≤0.06	2	32	≤0.125	64	16	>512	>512	512	4	*tetM*	*int-Tn*	
3	877	2	≤0.06	≤0.06	≤0.06	≤0.06	≤0.06	0.125	0.125	0.5	≤0.06	2	32	≤0.125	128	32	>512	>512	>512	4	*tetM*	*int-Tn*, *mobL*	
4	827KZ	2	≤0.06	≤0.06	≤0.06	≤0.06	≤0.06	0.125	0.125	0.5	≤0.06	2	32	8	128	32	>512	>512	>512	4	*tetM*	*int-Tn*	
5	827NW	2	≤0.06	≤0.06	≤0.06	≤0.06	≤0.06	0.125	0.125	0.25	≤0.06	2	16	8	16	32	>512	>512	>512	4	*tetM*	*int-Tn*	
6	1012	2	0.125	≤0.06	≤0.06	≤0.06	0.25	0.25	2	1	0.125	1	16	≤0.125	64	128	>512	>512	>512	4	*tetM*	*int-Tn*, *mobL*	
7	1023	2	≤0.06	≤0.06	≤0.06	≤0.06	≤0.06	32	1	>64	2	>64	32	16	>512	256	>512	>512	>512	4	*tetM*, *lnuB*, *lsaE*, *ant(6)-Ia*, *spw*, *erm47*	*int-Tn*, *virB*, *mobL*	
8	267	5	≤0.06	≤0.06	≤0.06	≤0.06	≤0.06	>32	>32	>64	>16	2	32	8	128	32	512	>512	>512	4	*tetM*, *ermB*	*int-Tn*	
9	86	5	≤0.06	≤0.06	≤0.06	≤0.06	≤0.06	0.125	0.125	>64	4	>64	64	8	>512	>512	>512	>512	>512	4	*tetM*, *lnuB*, *lsaE*, *ant(6)-Ia*, *spw*	*int-Tn*, *mobL*	ND
10	136	5	≤0.06	≤0.06	≤0.06	≤0.06	≤0.06	0.125	≤0.06	4	0.125	0.5	64	8	>512	256	>512	>512	>512	4	*tetM*, *ant(6)-Ia*, *spw*	*int-Tn*, *mobL*	rec *
11	147	5	≤0.06	≤0.06	≤0.06	≤0.06	≤0.06	0.125	0.125	8	≤0.06	0.5	32	8	>512	512	>512	>512	>512	4	*tetM*, *ant(6)-Ia*, *spw*	*int-Tn*, *mobL*	rec
12	413	5	≤0.06	≤0.06	≤0.06	≤0.06	≤0.06	0.25	0.125	0.25	≤0.06	1	32	8	64	64	>512	>512	>512	4	*tetM*	*int-Tn*	
13	512	5	≤0.06	≤0.06	≤0.06	≤0.06	≤0.06	0.125	0.125	0.5	≤0.06	2	32	8	64	32	>512	>512	>512	4	*tetM*	*int-Tn*	
14	652	5	≤0.06	≤0.06	≤0.06	≤0.06	≤0.06	0.125	0.125	0.25	≤0.06	1	32	8	32	32	512	>512	>512	4	*tetM*	*int-Tn*	
15	815	5	0.125	≤0.06	≤0.06	≤0.06	≤0.06	0.125	0.125	0.5	0.25	1	32	8	128	64	512	>512	>512	4	*tetM*	*int-Tn*, *mobL*	
16	1154	5	≤0.06	≤0.06	≤0.06	≤0.06	≤0.06	0.25	0.125	1	0.125	4	32	8	64	64	512	>512	>512	2	*tetM*	*int-Tn*	
17	43	5	0.125	≤0.06	≤0.06	≤0.06	≤0.06	0.125	0.25	0.5	≤0.06	1	32	8	64	16	>512	>512	>512	4	*tetM*	*int-Tn*	
18	44	5	0.125	≤0.06	≤0.06	≤0.06	≤0.06	0.125	0.25	0.5	≤0.06	2	32	8	64	64	>512	>512	>512	4	*tetM*	*int-Tn*	
19	1173	5	≤0.06	≤0.06	≤0.06	≤0.06	≤0.06	0.125	0.125	0.25	≤0.06	0.5	32	8	32	16	256	>512	>512	4	*tetM*	*int-Tn*	
20	13	5	0.125	≤0.06	≤0.06	≤0.06	≤0.06	0.25	0.125	0.5	≤0.06	2	32	8	128	64	>512	>512	>512	4	*tetM*	*int-Tn*, *mobL*	
21	48W	5	≤0.06	≤0.06	≤0.06	≤0.06	≤0.06	0.125	0.125	0.25	≤0.06	2	32	8	32	16	512	>512	>512	8	*tetM*	*int-Tn*	
22	51W	5	≤0.06	≤0.06	≤0.06	≤0.06	≤0.06	0.125	0.125	0.25	0.125	2	16	8	32	16	512	>512	>512	4	*tetM*	*int-Tn*	
23	89W	5	≤0.06	≤0.06	≤0.06	≤0.06	≤0.06	0.125	0.25	0.5	≤0.06	4	32	8	128	32	>512	>512	>512	4	*tetM*	*int-Tn*	
24	90W	5	≤0.06	≤0.06	≤0.06	≤0.06	≤0.06	0.25	0.25	0.5	≤0.06	4	32	8	64	32	>512	>512	>512	4	*tetM*	*int-Tn*	
25	936W	5	≤0.06	≤0.06	≤0.06	≤0.06	≤0.06	0.125	0.5	0.25	≤0.06	1	16	8	32	16	512	>512	>512	4	*tetM*	*int-Tn*	
26	434W	5	0.125	≤0.06	≤0.06	≤0.06	≤0.06	0.125	0.125	0.25	≤0.06	0.5	32	8	32	32	>512	>512	>512	4	*tetM*	*int-Tn*	
27	959	5	≤0.06	≤0.06	≤0.06	≤0.06	≤0.06	0.25	0.125	0.5	≤0.06	8	1	≤0.125	128	32	512	>512	>512	4			
28	584	5	≤0.06	≤0.06	≤0.06	≤0.06	≤0.06	0.25	0.125	0.5	≤0.06	2	1	≤0.125	64	32	512	512	>512	2			
29	803	5	≤0.06	≤0.06	≤0.06	≤0.06	≤0.06	0.25	0.25	1	≤0.06	1	1	≤0.125	128	64	>512	512	>512	2			
30	155	5	≤0.06	≤0.06	≤0.06	≤0.06	≤0.06	0.25	0.125	0.5	≤0.06	4	16	16	32	16	512	>512	>512	4	*tetM*	*int-Tn*	
31	95	8	≤0.06	≤0.06	≤0.06	≤0.06	≤0.06	0.125	0.125	>64	1	>64	32	16	64	32	512	>512	>512	8	*tetM*, *lnuB*, *lsaE*	*int-Tn*	rec, mcp **, mtp ***
32	489	8	≤0.06	≤0.06	≤0.06	≤0.06	≤0.06	0.125	0.125	>64	1	64	32	8	16	32	512	>512	>512	4	*tetM*, *lnuB*, *lsaE*	*int-Tn*	rec, mcp, mtp
33	176	8	0.125	≤0.06	≤0.06	≤0.06	≤0.06	0.25	0.125	>64	4	>64	64	8	64	32	>512	>512	>512	4	*tetM*, *lnuB*, *lsaE*	*int-Tn*	rec, mcp, mtp
34	670	8	≤0.06	≤0.06	≤0.06	≤0.06	≤0.06	0.125	0.125	>64	2	>64	32	8	128	64	>512	>512	>512	8	*tetM*, *lnuB*, *lsaE*	*int-Tn*	rec, mcp, mtp
35	759W	8	≤0.06	≤0.06	≤0.06	≤0.06	≤0.06	0.125	0.125	>64	2	64	32	8	128	32	>512	>512	>512	8	*tetM*, *lnuB*, *lsaE*	*int-Tn*	rec, mcp, mtp
36	818	8	0.125	≤0.06	≤0.06	≤0.06	≤0.06	0.125	0.125	>64	2	>64	32	8	64	32	>512	>512	>512	4	*tetM*, *lnuB*, *lsaE*	*int-Tn*	rec, mcp, mtp
37	819	8	0.125	≤0.06	≤0.06	≤0.06	≤0.06	0.125	0.125	>64	2	>64	32	8	64	32	>512	>512	>512	4	*tetM*, *lnuB*, *lsaE*	*int-Tn*	rec, mcp, mtp
38	1092	8	≤0.06	≤0.06	≤0.06	≤0.06	≤0.06	0.125	0.125	0.5	≤0.06	4	32	16	64	64	>512	>512	>512	4	*tetM*	*int-Tn*	
39	53	8	0.125	≤0.06	≤0.06	≤0.06	≤0.06	0.125	0.125	0.5	≤0.06	8	32	8	64	32	>512	>512	>512	8	*tetM*	*int-Tn*	
40	579	1b	0.125	≤0.06	≤0.06	≤0.06	≤0.06	0.25	0.125	0.5	≤0.06	2	32	8	128	32	>512	>512	>512	4	*tetM*	*int-Tn*	
41	167	1b	0.125	≤0.06	≤0.06	≤0.06	≤0.06	0.125	0.125	0.25	≤0.06	2	32	16	32	32	512	>512	>512	4	*tetM*	*int-Tn*	
42	395	1b	0.125	≤0.06	≤0.06	≤0.06	≤0.06	0.25	0.125	0.5	≤0.06	4	32	8	128	64	>512	>512	>512	8	*tetM*	*int-Tn*	
43	784	1b	0.125	≤0.06	≤0.06	≤0.06	≤0.06	0.125	0.125	0.25	≤0.06	2	32	8	64	32	512	>512	>512	4	*tetM*	*int-Tn*	
44	1042	1b	0.125	≤0.06	≤0.06	≤0.06	≤0.06	0.25	0.125	0.5	≤0.06	2	32	8	128	64	>512	>512	>512	4	*tetM*	*int-Tn*	
45	1228	1b	0.125	≤0.06	≤0.06	≤0.06	≤0.06	0.5	0.125	1	≤0.06	2	16	8	128	64	512	>512	>512	4	*tetM*	*int-Tn*	
46	439W	1b	≤0.06	≤0.06	≤0.06	≤0.06	≤0.06	0.125	0.125	0.5	≤0.06	2	32	8	64	32	>512	>512	>512	4	*tetM*	*int-Tn*	
47	450W	1b	≤0.06	≤0.06	≤0.06	≤0.06	≤0.06	0.125	0.125	0.25	≤0.06	1	32	8	64	16	512	>512	>512	4	*tetM*	*int-Tn*	
48	195	1b	0.125	≤0.06	≤0.06	≤0.06	≤0.06	0.25	0.25	2	0.125	4	1	≤0.125	128	64	>512	>512	>512	4			
49	657	1b	≤0.06	≤0.06	≤0.06	≤0.06	≤0.06	0.125	0.125	0.5	≤0.06	2	≤0.25	≤0.125	64	32	>512	>512	>512	4			
50	1083	1b	≤0.06	≤0.06	≤0.06	≤0.06	≤0.06	0.125	0.125	0.5	≤0.06	2	≤0.25	≤0.125	64	32	>512	>512	>512	4			
51	202	1b	≤0.06	≤0.06	≤0.06	≤0.06	≤0.06	0.25	0.125	1	0.125	4	32	8	128	64	>512	>512	>512	8	*tetM*	*int-Tn*	
52	525	1b	0.25	≤0.06	≤0.06	≤0.06	≤0.06	0.25	0.125	0.5	≤0.06	4	32	8	128	64	>512	>512	>512	4	*tetM*	*int-Tn*	
53	526	1b	0.125	≤0.06	≤0.06	≤0.06	≤0.06	0.25	0.125	1	≤0.06	4	16	16	128	64	512	>512	>512	4	*tetM*	*int-Tn*	
54	783	1b	0.125	≤0.06	≤0.06	≤0.06	≤0.06	0.25	0.125	1	0.125	8	64	16	64	32	>512	>512	>512	4	*tetM*	*int-Tn*	
55	633	1b	≤0.06	≤0.06	≤0.06	≤0.06	≤0.06	≤0.06	≤0.06	0.25	≤0.06	2	32	8	64	32	>512	>512	>512	4	*tetM*	*int-Tn*	
56	451	1b	0.125	≤0.06	≤0.06	≤0.06	≤0.06	0.25	0.125	0.5	≤0.06	2	32	16	128	64	>512	>512	>512	4	*tetM*	*int-Tn*	
57	49W	N	≤0.06	≤0.06	≤0.06	≤0.06	≤0.06	0.125	0.125	0.25	≤0.06	1	≤0.25	≤0.125	64	32	512	>512	>512	4			
58	320	N	0.125	≤0.06	≤0.06	≤0.06	≤0.06	0.25	0.125	0.5	≤0.06	4	32	8	128	64	>512	>512	>512	4	*tetM*	*int-Tn*	
59	497	6	0.125	≤0.06	≤0.06	≤0.06	≤0.06	0.25	0.125	0.5	≤0.06	2	32	16	128	64	>512	>512	>512	4	*tetM*	*int-Tn*	
60	849	6	≤0.06	≤0.06	≤0.06	≤0.06	≤0.06	≤0.06	0.125	0.25	≤0.06	1	≤0.25	≤0.125	32	16	512	512	>512	4			
			0	0	0	0	0	2 3.3%	3 5%	10 16.7%	10 16.7%	9 15%	51 85%	48 80%	ND	ND	ND	ND	ND	ND			

Legend: MIC values highlighted in grey indicate resistance, and those highlighted in blue indicate medium susceptibility. Ser.—serotype; PEN—penicillin; AMP—ampicillin; AMX—amoxicillin; AMC—amoxicillin + ca; CEF—ceftiofur; TET—tetracycline; ERY—erythromycin; TYL—tylosin; CLI—clindamycin; LIN—lincomycin; TIA—tiamulin; ENR—enrofloxacin; STR—streptomycin; SPE—spectinomycin; GEN—gentamicin; NEO—neomycin; TR/S—trimethoprim/sulfamethoxazole; FLO—florfenicol; * rec—gene coding for recombinase family protein; ** mcp—gene coding for phage major capsid protein; *** mtp—gene coding for phage minor tail protein.
